# Using direct clinical observation to assess the quality of cesarean delivery in Afghanistan: an exploratory study

**DOI:** 10.1186/1471-2393-14-176

**Published:** 2014-05-27

**Authors:** Cherrie Lynn Evans, Young Mi Kim, Khalid Yari, Nasratullah Ansari, Hannah Tappis

**Affiliations:** 1 Jhpiego/USA, an affiliate of Johns Hopkins University, 1615 Thames St., Baltimore MD 21231, USA; 2 Jhpiego/Afghanistan, an affiliate of Johns Hopkins University, Kabul, Afghanistan; 3 UNICEF, UNOCA Compound, Jalalabad Rd., Kabul, Afghanistan; 4 Department of International Health, Johns Hopkins Bloomberg School of Public Health, 615 N. Wolfe St., Baltimore MD 21205, USA

## Abstract

**Background:**

As part of a National Emergency Obstetric and Newborn Care (EmONC) Needs Assessment, a special study was undertaken in July 2010 to examine the quality of cesarean deliveries in Afghanistan and examine the utility of direct clinical observation as an assessment method in low-resource settings.

**Methods:**

This cross-sectional assessment of the quality of cesareans at 14 facilities in Afghanistan included a survey of surgeons regarding their routine cesarean practices, direct observation of 29 cesarean deliveries and comparison of observations with facility records for 34 additional cesareans conducted during the 3 days prior to the observation period at each facility. For both observed cases and record reviews, we assessed time intervals between specified points of care-arrival to the ward, first evaluation, detection of a complication, decision for cesarean, incision, and birth.

**Results:**

All time intervals with the exception of “decision to skin incision” were longer in the record reviews than in observed cases. Prior cesarean was the most common primary indication for all cases. All mothers in both groups observed survived through one hour postpartum. Among newborns there were two stillbirths (7%) in observed births and seven (21%) record reviews. Although our sample is too small to show statistical significance, the difference is noteworthy. In six of the reviewed cesareans resulting in stillbirth, a fetal heart rate was recorded in the operating theater, although four were recorded as macerated. For the two fresh stillbirths, the cesarean surgeries were recorded as scheduled and not urgent.

**Conclusions:**

Direct observation of cesarean deliveries enabled us to assess a number of preoperative, postoperative, and intraoperative procedures that are often not described in medical records in low resource settings. Comparison of observations with findings from provider interviews and facility records allowed us to infer whether observed practices were typical of providers and facilities and detect potential Hawthorne effects.

## Background

Worldwide, an estimated 258,000 women die each year from pregnancy-related causes, with 99% of these deaths occurring in developing countries. Of these deaths, 87% occur in South Asia and sub-Saharan Africa, where progress in reducing these deaths is slow with declines of 1% per year or less. While there have been recent gains, an estimated 6,400 women in Afghanistan still die each year from pregnancy-related causes. Maternal mortality is estimated at 460 maternal deaths per 100,000 live births [1].

Adequately functioning health systems are necessary on multiple levels to ensure the survival of mothers and newborns, including quality community-level interventions, functioning referral mechanisms, and sufficiently resourced facilities [2-4]. In this paper, we focus on facility-based delivery, and in particular on cesarean delivery. While clearly not the only important obstetric procedure or skill necessary to improve maternal and perinatal survival, cesarean delivery is an indispensable intervention for saving lives. The recommended lower and upper limits for cesarean delivery in any population are often quoted at 5% and 15% respectively, but there is no empirical evidence that these rates are optimal. Both very low and very high rates of cesarean delivery can be problematic, the former because they may reflect women’s lack of access to life-saving care for complications such as obstructed labor or placenta previa, and the latter because they may indicate overuse of the surgical procedure, which carries with it increased risk during and after surgery [5,6]. Therefore, cesarean delivery should be used when the benefit to the mother or fetus is clear. Particularly in a conflict-affected, low-resource country such as Afghanistan where 67% of women give birth at home and 61% without a skilled attendant and cesarean delivery rates are only 3.6%, poor access to facilities may put women out of reach of emergency care during subsequent pregnancies when their history of prior uterine scar puts them at higher risk [7]. In this context, appropriate and judicious use of cesarean delivery is even more important.

As part of a National Emergency Obstetric and Newborn Care (EmONC) Needs Assessment conducted in 2009, a special study was undertaken to examine cesarean delivery in Afghanistan. Components of the broader assessment of the availability and quality of EmONC services examined contributing factors to quality of cesarean delivery. The first phase of the assessment included applying the UN Process Indicators in order to assess availability, quality, and utilization of EmONC, including cesarean delivery, and the evaluation of structural indicators (infrastructure, utilities, equipment, supplies, drugs, staffing, etc.) that affect the ability to provide obstetric care. Briefly, these analyses found that only 79% of the 78 government facilities designated to provide cesarean deliveries had done so in the three months preceding the assessment, and that reasons for not providing this life-saving service included lack of human resources; management issues; lack of supplies, drugs and equipment; and lack of training. Detailed findings are reported elsewhere [8]. This assessment helped us to understand what was happening at the system level, but could not provide necessary detail to explain what was happening at the point of care, particularly in regards to cesarean service delivery. While system-level assessments are important for improving various aspects of service delivery, alone they are insufficient.

A second phase of the assessment was added to examine cesarean delivery by reviewing charts from the three most recent cesareans at the 62 facilities assessed that had conducted cesarean deliveries in the three months before the study. This phase of the study identified multiple deficits of quality of care which may have contributed to the large number of maternal and fetal deaths reported in the sample of cases reviewed, including limited use of a partogram to monitor labor and delays between the decision to conduct a cesarean delivery and initiation of the procedure [9]. In addition, we found that many records were missing key information on the process and timeliness of care. Study findings from both phase 1 and phase 2 of the assessment highlighted the need for a more in-depth examination of the quality of cesarean deliveries in Afghanistan, and prompted the addition of a third phase of the study involving direct observations of the surgical process which is the subject of this paper.

Good cesarean delivery practices require not only technical competence but also appropriate and timely decision-making. A variety of methods have been employed to assess the process of care such as criterion based audits, a form of clinical audit that seeks to improve patient care and outcomes through systematic review of records against explicit criteria, and implementation of changes with further monitoring to confirm improvement in healthcare delivery. Criterion-based audits have been shown to improve obstetric practice and health outcomes where baseline adherence to quality standards is poor [10]. Although this method is an increasingly common and potentially powerful approach to measuring quality of care, good record keeping is key to the validity of any assessment as audits assume that what is recorded was actually performed and what was not recorded was not performed [11]. Because we identified missing information in the clinical record we chose to directly observe care of women undergoing cesarean delivery.

Direct clinical observation (DCO) is an assessment method that has been used to evaluate the actual process of clinical care. Most research using this method in developing countries has been done in outpatient settings evaluating patients with common medical complaints such as fever and diarrhea [12,13]. Das and colleagues also observed outpatient prenatal care. In an inpatient setting, Burkhalter and colleagues used DCO to identify deficiencies in the quality of routine labor and delivery care in labor wards in four developing countries [14]. However, reports of using direct observation to assess the quality of surgical care outside of training are limited. The objectives of this study were to assess the feasibility and usefulness of DCO for assessment of cesarean deliveries in low-resource settings and to collect first-hand information on the timeliness of decision-making, provision of care and outcomes of cesarean deliveries that could address gaps or be triangulated with findings from other studies on EmONC service provision in Afghanistan.

## Methods

### Study setting

A cross-sectional descriptive assessment of the quality of cesarean delivery in Afghanistan was conducted during July 2010. Of the 127 health facilities designated as comprehensive emergency obstetric and neonatal care (CEmONC) facilities in Afghanistan’s 34 provinces (first line referral facilities expected to provide parenteral antibiotics, parenteral anticonvulsants, parenteral uterotonics, manual removal of placenta, removal of retained products, assisted or instrumental vaginal delivery, newborn resuscitation, blood transfusion and cesarean surgery), only 26 performed an average of 10 or more cesarean surgeries per week in the preceding year. Twelve of these facilities were inaccessible due to security constraints at the time of the assessment. The study sample consisted of the remaining 14 accessible facilities that performed an average of 10 or more cesarean surgeries per week: four Specialized Hospitals (SHs), three Regional Hospitals (RHs), and seven Provincial Hospitals (PHs). In these facilities we observed 29 cesarean deliveries from the pre-operative period through one hour after surgery, including immediate postpartum and newborn care (Additional file 1), and reviewed records of an additional 34 cesarean deliveries conducted within 60 hours before the assessment team arrived (Additional file 2). We also interviewed the provider from each of these cases (Additional file 3) and provided self-administered questionnaires on practices related to cesarean delivery to these providers and all other obstetric surgeons at each study facility (Additional file 4). We received responses to these questionnaires from all 83 surgical providers working at the study facilities.

### Instrument development

Tools were developed or adapted to document: 1) facility readiness to perform cesarean surgeries; 2) clinical observations of cesarean surgery and post-operative and newborn care; 3) cesarean delivery case management and outcomes recorded in facility medical records and operating theater logbooks; 4) provider perspectives on the indications for surgery, urgency of surgery and choice of anesthesia; and 5) surgical providers’ training experiences and usual practices during and after cesarean delivery.

Clinical observations of cesarean surgery, post-operative care and newborn care were documented with a tool based on the Jhpiego Standards-Based Management and Recognition (SBM-R) checklist for cesarean delivery. SBM-R is a quality assurance method focused on the standardization and implementation of best practices. The SBM-R checklist for cesarean delivery is much lengthier than the clinical observation tool used in this study; the quality assurance tool was shortened considerably to include only a sub-set of items considered most important for good-quality care, in accordance with the WHO Integrated Management of Pregnancy and Childbirth manual *Managing Complications of Pregnancy and Childbirth*[15].

Details of the three most recent cesarean deliveries reported in medical records and operating theater logbooks as taking place between 12 and 60 hours before arrival of the data collectors were documented using a structured checklist designed to collect case information and outcomes for comparison with observed deliveries. Surgical provider reports of the indications for cesarean delivery and urgency of surgery and anesthesia provider choices of anesthesia for each case observed during the assessment and documented in the record review were collected using structured questionnaires developed for this purpose. Finally, all obstetric surgical providers at each facility visited were invited to take a short, self-administered questionnaire reviewing their training, confidence, and customary practices regarding cesarean delivery. All instruments were reviewed by specialist obstetricians and midwives at Jhpiego in Baltimore and Afghanistan, and are provided as supplementary materials to accompany this manuscript.

### Data collection

The assessment team consisted of 15 Afghan obstetricians/gynecologists and 3 Afghan midwives, all trained in direct clinical observation and chart review. The data collectors attended three days of training on the study methodology and data collection instruments, as well as on research ethics, data storage, and confidentiality. Data collection took one to two days per facility, depending on the cesarean delivery caseload. Facilities were not informed in advance about the assessment team’s visit. When the data collectors arrived, written informed consent was obtained from the heads of the units where cesarean deliveries were conducted. Oral informed consent was obtained from providers and patients before any observation began. The study team began observation with the first cesarean delivery that took place after arrival. No identifying data were collected about the surgical providers conducting cesarean deliveries or women undergoing surgery. To maintain privacy, all observations and interviews were carried out in private rooms or spaces. Medical records and operating theater logbooks were reviewed for the three most recent cesarean deliveries occurring from 12 to 60 hours before the study team’s arrival; if less than three cesarean deliveries occurred during this time period, all records were reviewed. All obstetric surgical providers and anesthesia providers involved in the 29 cases observed and included in the record review were invited to participate in interviews. All 83 obstetric surgical providers present at the facility during the assessment team’s visit were also invited to complete self-administered questionnaires. This study received approval from institutional review boards at the Afghanistan Public Health Institute (IRB #2333) and Johns Hopkins Bloomberg School of Public Health (IRB #2359).

## Results

During three weeks in July 2010, a total of 29 cesarean deliveries were observed and records from 34 additional cesarean deliveries were reviewed in 14 hospitals.

Four (14%) of the cesarean deliveries observed were women transferred from lower level hospitals facilities. The remaining 25 (86%) presented from home. Two-thirds (66%, n = 19) presented for routine labor, and the remainder (n = 10) presented with a complication. Nearly half (41%, n = 12) of women were grand multiparas (more than four prior births), and only 17% were primiparas (no prior births). The majority of cesarean deliveries observed were performed by an obstetrician/gynecologist (66%, n = 19), with anesthesia either provided by anesthesiologists (52%, n = 15) or by anesthesia nurses or technicians (48%, n = 14).

Seven (21%) of the cesarean delivery case records reviewed were from women transferred from lower level facilities and 27 (79%) were women presenting from home. Of the 29 case records reporting parity, 17 (59%) were grand multiparas and 8 (28%) were primiparas. Of the 28 case records reporting the type of clinician that performed the cesarean, 20 (71%) were performed by an obstetrician gynecologist. Ten (34%) of the 29 cesarean deliveries observed and 14 (41%) of the 34 cesarean delivery case records reviewed arrived at the hospital with a complication that had been detected at another facility or experienced at home with the remainder presenting for routine labor.

### Timing

For both observed cases and record reviews, we assessed time intervals between specified points of care—the woman’s arrival, the first evaluation, the detection of complications, the decision for cesarean delivery, the incision, and birth. The average time elapsed between events for women admitted to the hospital for routine labor and those admitted with obstetric complications already evident is presented in Table 1. All time intervals with the exception of “decision to skin incision” were longer in the record reviews than in the observed cases.

**Table 1:** Average time between events (hours: minutes) for women undergoing caesarean deliveries

	**Women admitted for routine labor**		**Women admitted for a complication**	
	**Observed (n = 19 )**	**Record review (n = 20 )**	**Observed (n = 10 )**	**Record review (n = 14)**
From arrival to first evaluation	00:11	00:17	00:03	00:19
From arrival to complication detected	02:01	06:24	00:58	02:27
From complication detected to decision for CS	00:24	01:00^a^	01:03	00:31
From decision for CS to skin incision	01:11	00:58^a^	00:40	00:51^b^
From skin incision to delivery	00:07	00:16^a^	00:07	00:08 ^b^
*Total*	*07:54*	*08:55*	*02:51*	*04:16*

^a^One record did not list time of decision for CS, incision or delivery.

^b^One record did not list time of incision or delivery, and two did not list time of delivery.

For all observed cases, the average time from arrival at the facility to first evaluation by a clinician was eight minutes (range 0 minutes – 1 hour, 30 minutes). The average time elapsed from arrival to first evaluation was 3 minutes for those admitted with complications and 11 minutes for those admitted for routine labor. Complications needing to be addressed by cesarean delivery were detected an average of 1 hour, 40 minutes (range 0 minutes – 17 hours, 30 minutes) after arrival, approximately one hour after arrival in cases presenting with a complication and two hours after arrival in cases presenting with routine labor. The average time elapsed between detection of a complication to the decision to conduct a cesarean delivery for all observed cases was 39 minutes (0 minutes – 1 hour, 35 minutes). The decision to conduct a cesarean delivery occurred an average of 1 hour, 3 minutes after the indicative complication was detected in women arriving at the facility with obstetric complications and 24 minutes after the indicative complication was detected for women admitted for routine labor. The average time from decision to conduct a cesarean delivery to skin incision for all observed cases was 1 hour (range 10 minutes – 4 hours, 5 minutes), and average time from detection to delivery was an average of 1 hour, 50 minutes for women arriving at the facility with obstetric complications and 1 hour, 42 minutes for those admitted for routine labor.

### Indications for cesarean delivery

Primary indications reported by surgical providers are presented in Table 2. The most common primary indication for cesarean delivery in the observed cases was prior uterine scar, followed by fetal distress and then prolonged labor. Of the four women with prolonged labor as the indication for cesarean delivery, only one had labor followed with a partogram, and none had labor pharmacologically augmented. Provider interviews for each case revealed that the cesarean surgery was considered an emergency in 76% of observed cases. For four emergency cases, the primary indication was prior cesarean and for one it was meconium stained fluid. Among the case records reviewed, prior uterine scar was also the most common primary indication for cesarean delivery, followed by malpresentation, and then placenta previa.

**Table 2:** Primary indication reported by surgical provider for caesarean deliveries

	**Observed (n = 29)**	**Record review (n = 34 )**
History of uterine scar	24%	21%
Fetal distress indicated by abnormal fetal heart beat	17%	6%
Failure to progress in labor/obstructed labor	14%	12%
Malpresentation of the fetus	10%	18%
Suspected uterine rupture	7%	0%
Oligohydramnios	7%	0%
Placenta previa	3%	12%
Vaginal hemorrhage	3%	0%
Meconium stained amniotic fluid	3%	2%
Pre-eclampsia/eclampsia	3%	2%
Other or multiple indications	7%	18%
Missing data*	N/A	9%
*Total*	98%*	*100%*

*Column does not sum to 100% due to rounding.

### Operative procedures in observed cesarean deliveries

Operative procedures conducted during observed cesarean deliveries are presented in Table 3. In all observed cases an IV line and a urinary catheter were inserted prior to surgery. However, in only 46% of cases was the vulva cleansed before insertion of the catheter. Only 14% received a gastric acid inhibitor or neutralizer to decrease the risk of morbidity associated with aspiration of stomach contents during surgery. In 21% of cases mothers were placed in a lateral tilt on the operating table, a simple technique which is often used to prevent a decrease in blood supply to the fetus from compression of maternal blood vessels and hypotension. Low transverse skin incision, which is often done for cosmetic purposes but is not recommended for emergencies [15] was used in 34% of cases. A low transverse uterine incision, which decreases the risk of uterine rupture in subsequent pregnancies, was used in all observed cases.

**Table 3:** Percent of observed caesarean deliveries with the indicated tasks performed during the operative period

	% of cases*
IV line inserted	100%
Vulva cleansed prior to insertion of urinary catheter (n = 28)	46%
Urinary catheter inserted	100%
Any antacid or acid neutralizer given	14%
Lateral tilt of mother on table	21%
Fetal heart assessed in the operating theater (n = 27)	67%
Regional anesthesia used	72%
Check for adequate anesthesia prior to incision (n = 26)	92%
Low transverse skin incision	34%
Low transverse uterine incision	100%
Maternal prophylactic antibiotics after cord clamping	90%
Closed the peritoneum	45%
Tubal ligation performed (n = 28)	32%
Final sponge/instrument count	64%
Blood pressure and pulse monitored at least every 15 minutes during surgery	83%

*n = 29 unless otherwise noted due to missing data.

In 72% of observed cases, providers chose to use regional anesthetic; general anesthesia was used for the remainder. In the eight cases where general anesthesia was used, half of providers reported choosing that method because the surgery was considered an emergency. In the other half the providers said that they chose general anesthesia because it was “safe” or “more comfortable.”

### Outcomes of observed and reviewed cesarean deliveries

All mothers in both the observed and record review arms survived through two hours postpartum. Among newborns there were two stillbirths (7%) in the 29 observed births, both of which occurred after 6:30 pm. There were seven stillbirths (21%) in the 34 case records reviewed, two of which were fresh stillbirths that occurred after 4:00 pm. Statistical analysis using Fisher’s Exact Test did not show this to be a significant difference at the p < 0.05 level. In the observed group there was one fresh stillbirth, resulting from a cesarean delivery for prolonged labor, and one macerated stillbirth in a cesarean done for hemorrhage. No assessment of the fetal heart was performed in the operating theater nor was attempted resuscitation of the newborn observed in either case. In six of the case records reviewed, a fetal heart beat was recorded in the operating theater, although four of these stillbirths were recorded as macerated. Three of these seven cesarean sections were recorded as “scheduled, not due to an emergency.”

### Routine cesarean delivery practices reported by surgical providers

Eighty-three providers were invited to fill in the questionnaire on routine practices, and all responded. Five of the questionnaires had significant data missing, however, and were not used in the analysis. Responding surgical providers had performed an average of 36 cesarean deliveries in the preceding six months, with a range from 0 to 200. Of these providers 58% said they were “very confident” about performing cesarean sections. Only 4% expressed a lack of confidence. Routine practices that meet international standards and those that are usually considered unnecessary and potentially harmful are presented in Table 4.

**Table 4:** Routine caesarean delivery practices reported by surgical providers

	% of providers*
***Practices that are usually considered unnecessary and potentially harmful***	
Administer enema prior to cesarean delivery	3%
Shave the abdomen and public hair prior to cesarean	3%
Open the uterine cavity by vertical incision (n = 76)	4%
Close the peritoneum (n = 77)	32%
Routinely transfuse one unit of blood during or after cesarean (n = 77)	18%
Routinely perform tubal ligation after the mother’s third cesarean delivery (n = 77)	25%
Restrict oral intake for 24 hours postoperatively	94%
Restrict ambulation for 24 hours postoperatively	29%
Prescribe antibiotics for 5 to 7 days in all postoperative cases (n = 76)	71%
Routinely conduct cesarean delivery for women who have eclampsia (n = 69)	21%
Routinely conduct cesarean delivery for women who are considered short in stature and are primigravidas (n = 76)	28%
Routinely conduct cesarean delivery for women who have had one prior cesarean delivery (n = 76)	18%
Routinely conduct cesarean delivery for women who have meconium-stained fluid in early labor (n = 77)	42%
***Practices that are considered appropriate for routine care***	
Use regional anesthesia (n = 73)	68%
Open the abdomen using transverse incision (n = 77)	70%
Routinely conduct cesarean delivery for women who have had two prior cesarean deliveries	82%

*n = 78 unless otherwise noted due to missing data.

Surgeons were asked what indications routinely led them to perform cesarean delivery. Eighty two percent stated that they perform cesarean routinely for women who have had more than one prior cesarean delivery. Forty-two percent considered the presence of meconium in amniotic fluid to be an indication for cesarean delivery.

More than two-thirds (68%) of the providers said that they prefer to use regional anesthesia for cesarean sections. Seventy percent typically perform transverse incision on the skin, and 96% use transverse incision also on the uterus. Eighteen percent routinely transfuse one unit of blood when performing a cesarean section. Some 86% of surgeons give one dose of antibiotics prophylactically to all women undergoing cesarean delivery, as recommended (data not shown), although 71% continue antibiotics routinely during the first post-surgical week, which is not recommended.

## Discussion

Direct clinical observation of cesarean deliveries enabled us to assess a number of preoperative, postoperative, and intraoperative procedures that are often not described in medical records in low resource settings. Observing cases allowed us to evaluate timeliness of provider decision-making, performance of surgical operations, and routine practices in the pre- and post-operative period that affect quality of care for women with obstetric complications who were delivered by cesarean. Comparison of observed findings with case information contained in medical records for cesarean deliveries conducted between 12 and 60 hours before the assessment team’s arrival at each facility, as well as both facilitated and self-administered interviews with surgical providers allowed us to identify potential Hawthorne effects where study subjects may have changed their behavior because they knew they were being observed.

It is widely recognized that decreasing the time between presentation with an obstetric complication and treatment is crucial to improving outcomes [16,17]. Women who present for normal labor also must be evaluated in a timely fashion and monitored appropriately throughout labor to detect any underlying or developing complications. The distinction between timing from arrival to first evaluation by a provider and to identification of a complication for women arriving at the hospital with a complication and those presenting with routine labor is important. One would assume that women arriving with a suspected complication would be evaluated more quickly through triage mechanisms. As for time to identification of a complication, women who arrive for care of routine labor may not develop a complication for some time. Once a complication is detected, however, one would expect similarity between the two groups in the next three time intervals—from detection of the complication to the decision for cesarean delivery, from that decision to the abdominal incision, and from incision to delivery. In the cases observed, the total time from detection of complications to delivery was similar for both groups (1 hour, 50 minutes for women admitted with complications and 1 hour, 40 minutes for women admitted for routine labor). However, the average time elapsed for specific intervals varied between the two groups. The first of these time intervals—from detection of a complication to the decision—was an average of 39 minutes longer for women admitted with a complication compared to those admitted for routine labor, while the second of these time intervals – from decision to conduct a cesarean delivery to skin incision – was an average of 31 minutes shorter for women admitted with a complication compared to those admitted for routine labor. These time differences do not reflect the nuances in clinical decision making for different complications. For example one would expect more urgency for a complication such as placenta previa with active hemorrhage than for prolonged labor without signs of obstruction or fetal distress.

For both women admitted with a complication and those admitted for routine delivery, the average time from arrival to first evaluation by a provider and from arrival to detection was shorter for observed cases than those included in the record review. For women presenting with complications, there are two possible explanations for the shorter time intervals in the observed group between arrival and first evaluation, and between arrival and detection of that complication. Either record-keeping may have been less accurate than observation, or else observed women were, in fact, attended to more quickly than the women who were not observed. Indeed, the Hawthorne effect may have contributed to the very short times between the arrival of women at the facility and their first evaluation. Whether considering time intervals or surgical practices, we assume that generally providers will be more likely to function at the higher end of their capacity while being observed. Thus, we can assume that care will not usually be better in unobserved cases but rather will be the same or possibly lower quality. Although our sample is too small to show statistical significance, the difference in the rates of stillbirth in the observed group (7%) and in the unobserved group (21%) is noteworthy. While medical records may not contain all pertinent information, we assume that, between the medical record and the interview with the surgeon, the method of delivery (whether vaginal or cesarean) and the status of the newborn (whether alive or dead) are accurately recorded in our study. If so, the Hawthorne effect may actually have produced a protective effect for newborns, and perhaps lack of motivation may have lead surgical providers to perform at less than their full capabilities when they were not being observed.

Average time elapsed during three critical time intervals – from arrival of women at the facility to detection of a complication, from detection of a complication to decision, and from decision to incision – in both the observed and record review groups were consistent with time intervals documented in the chart review conducted for phase two of the National EmONC Assessment [9] and highlight the need for improved monitoring and quality assurance measures such as establishment of a cesarean delivery surveillance system [18] or introduction of maternal death and near miss audits to identify when, where, and why delays occur at each facility.

In addition to timeliness of decision making, appropriate evaluation of clinical indications for cesarean delivery is critical for ensuring quality provision of lifesaving care. By self-report, we found a significant number of obstetric surgeons perform cesarean for meconium liquor in early labor. Meconium staining of the amniotic fluid is not uncommon at term and alone is not an indication for cesarean section. Nevertheless, one of the observed cases and one of the case records reviewed identified meconium stated amniotic fluid as the primary indication for surgery.

Direct clinical observation of cesarean deliveries and interviews with surgical providers enabled us to further assess the quality of a number of preoperative, postoperative, and intraoperative procedures that are often not described in medical records. Of the four women observed with prolonged labor as the indication for cesarean delivery, only one had her labor followed with a partogram, and none had labor pharmacologically augmented. This is also consistent with the chart review conducted for phase two of the National EmONC Assessment, which reported that only 28% of the cesarean delivery cases reviewed were managed with a partogram [9]. The partogram is a simple and inexpensive tool recommended by the World Health Organization for monitoring the progress of labor [19]. By graphically displaying progress of labor as a decision making tool, partogram use has been shown to decrease prolonged labor and reduce unnecessary cesarean delivery in low resource settings [19] Oxytocin augmentation was not used in any cesarean deliveries performed for prolonged labor. Appropriate use of pharmacologic augmentation can reduce the incidence of prolonged labor and thus the need for cesarean delivery. Four of the nine stillbirths (observed and unobserved cases) had a diagnosis of prolonged labor.

Other simple and inexpensive or free interventions that can improve quality of care in cesarean deliveries include basic infection control measures to prevent iatrogenic urinary tract infections, the use of a gastric acid neutralizer to decrease the risk of significant morbidity associated with aspiration during surgery, and use of the lateral tilt to take the weight of the uterus off the major blood vessels supplying the fetus. The vulva was cleansed before insertion of a urinary catheter in just 46% of observed cases. Only 14% received a gastric acid inhibitor or neutralizer to decrease the risk of morbidity associated with aspiration of stomach contents during surgery. In 21% of cases mothers were placed in a lateral tilt on the operating table. These simple interventions could be supported and highlighted nationally by pre-service educators, professional organizations, and facility administrators. A standardized pre-service and in-service training system should be established to fill the gaps of knowledge skills and attitude of surgical health care providers.

Direct clinical observation only provides information about the cases occurring during the assessment period and does not provide insight into whether the procedures conducted are typical of the providers or facilities. Facilitated and self-administered interviews with surgical providers provided additional information to address this gap. While some findings of the interviews were quite positive, others were more concerning. Prophylactic use of antibiotics (regardless of regimen) in women undergoing cesarean section reduces the risk of infection-related complications and serious infection post-operatively^7^. Our study found that 86% of women were given prophylactic antibiotics after clamping of the cord during cesarean delivery. We found, however, that more than three-fourths of providers routinely prescribed antibiotics to all cases for five to seven days after delivery. This practice is contrary to both national guidelines and international standards and should be addressed through pre-service education and in-service training. Supportive supervision, monitoring and reporting systems need to be improved and strengthened. Another quality issue in the delivery of cesarean section that surfaced in our study is routine blood transfusion during and after cesarean delivery. This practice, too, fails to meet Afghan and global blood transfusion standards. This unnecessary practice may expose women to blood-borne infections as well as to the risk of transfusion reaction.

### Strengths and limitations

With only 32% of deliveries in Afghanistan taking place in facilities, [7] this study provides insight to the care available to only a small proportion of the population. Still, the ability to give good-quality facility-based care and to provide quality care for cesarean delivery is one important component of providing lifesaving services for women and newborns. In addition, women are more likely to access facility based care if they know it is of high quality. Observing cases allowed us to assess provider performance and identify areas where provider knowledge, skills or decision-making may benefit from supportive interventions. In addition, conducting observations alongside a more detailed chart review than was conducted in previous studies, and interviews of providers about both the observed and unobserved cases, allowed us to make inferences about the possible biases associated with direct observation and triangulate findings to provide a stronger evidence-base for quality improvement recommendations.

Problems with security imposed the greatest limitations on the study. Of 127 designated EmONC facilities nationwide, the team could safely observe cesarean delivery at only 14 of the 26 facilities meeting study criteria thus limiting the numbers of cases observed and reviewed. This particularly affected our analysis of stillbirths between the two groups. The hospitals available for data collection in this phase were all tertiary hospitals; due to security concerns no district hospitals could be included. It is likely that recommendations stemming from this research will be even more important to the country’s lower-level first referral hospitals. In addition to this limitation, because the data collectors routinely arrived in the morning (in part due to security concerns), data from observed cases examines cesarean deliveries that typically were handled by the staff present in the morning. Of the 29 observed cases, only three occurred between 6:30 pm and 6:30 am with two of those cases ending in stillbirth. In the reviewed cases, seven occurred during those night hours and 9 had missing data for time. Of note, the two fresh stillbirths identified in the case records reviewed were delivered after 4:00 pm. Because of the availability of more human resources during daytime hours and the high levels of experience present, it is likely that what we observed is the higher end of functioning at these facilities compared with the reviewed cases which were more evenly spread throughout day and night.

## Conclusions

Direct clinical observation is a feasible, effective method for assessing quality of care and outcomes of cesarean deliveries in low-resource settings. However, direct clinical observation only provides information about the cases occurring during the assessment period and does not provide insight into whether the procedures conducted are typical of providers or facilities. Comparison of observations with findings from provider interviews and chart reviews can identify potential Hawthorne effects where study subjects may have changed their behavior because they knew they were being observed and suggest whether observations are typical of providers and facilities or not. Combining observation with record review and provider interviews can provide a more detailed assessment of strengths and weakness in quality in order to design targeted interventions to change practice.

## Competing interests

The authors declare that they have no competing interests. The study was one component of a national emergency obstetric and newborn care needs assessment funded by UNICEF and conducted by Jhpiego, an affiliate of Johns Hopkins University, in collaboration with the Ministry of Public Health of Afghanistan. Any opinions stated are those of the authors and not of UNICEF, Jhpiego or the Johns Hopkins Bloomberg School of Public Health.

## Authors’ contributions

CE was primary designer of the study protocol and study instruments and was the chief writer of the manuscript. YMK served as the Principal Investigator of the study and coordinated the manuscript drafting and finalization process. KY and NA participated in the design and implementation of the study, contributed to data analysis, and provided key input into the analysis, interpretation of results, discussion and conclusions. HT contributed to the writing of the results and discussion sections and to critical review and revision of the manuscript. All authors read and approved the manuscript.

## Pre-publication history

The pre-publication history for this paper can be accessed here:

http://www.biomedcentral.com/1471-2393/14/176/prepub

## Supplementary Material

Additional file 1 Afghanistan Emergency Obstetric and Newborn Care National Assessment Tool: Clinical Observation of Cesarean Section. Click here for file

Additional file 2 Afghanistan Emergency Obstetric and Newborn Care National Assessment Tool: Review of Unobserved Post-Cesarean Case. Click here for file

Additional file 3 Afghanistan Emergency Obstetric and Newborn Care National Assessment Tool: Interview of Anesthesia and Surgical Providers. Click here for file

Additional file 4 Afghanistan Emergency Obstetric and Newborn Care National Assessment Tool: Self-Administered Questionnaire of Surgical Provider’s Experience and Practice. Click here for file
