# Supplementary material for: Using direct clinical observation to assess the quality of cesarean delivery in Afghanistan: an exploratory study
Source: BMC Pregnancy Childbirth. 2014 May 27;14:176. doi: 10.1186/1471-2393-14-176 (PMC4062280; doi:10.1186/1471-2393-14-176)
Supplement: Additional file 1 — Afghanistan Emergency Obstetric and Newborn Care National Assessment Tool: Clinical Observation of Cesarean Section. [file 1471-2393-14-176-S1.pdf]

Approved: 15APR10

Hospital: – National ID Number: ____________________
Study-generated Surgical Provider ID: ____________________
Study-generated ID for the CS Case: ____________________

EmONC Needs Assessment in Afghanistan-Part III Cesarean Section
Jhpiego

Tool 2: Clinical Observation of Cesarean Section

~~~~~~

	Information about Data Collection	Response
1	Assessor Name /ID Number:	
2	Health Facility Type: *(circle response)*	DH(1), PH(2), RH(3), SH(4)
3	Today's Date: (mm/dd):	___/ ___
4	Study-generated Surgical Provider ID: _	

Instructions. There are three sections of this Observation Tool:

- *Section A. Preliminary Information About the Observation*
- *Section B. Observation of Cesarean Surgery*
- *Section C. Observation of Post-Operative and Newborn Care*

Please note that this tool should be followed by Tool 3: Interview with Surgical Provider on the Observed Case.

SECTION A - Preliminary Information About the Observation

Please obtain the information in Section A by asking the nurse or checking the medical chart. Please fill in the response in the space provided, or circle the appropriate number.

000	Provider Team Cadres	Response
001	What is the cadre or qualification of the…<br>…surgical provider? *(write in space)*	________________________
~~~~~~

002	…anesthesia provider?	___________________________
003	…nurse?	___________________________
004	(Continued…What is the cadre or qualification of the…)<br><br>… other team member?	___________________________

100	Woman's Characteristics and Arrival	Response
101	What is the age of the mother/patient?<br>*(write age or 99 for not recorded)*	______
102	What is the parity of the mother?<br>*(write age or 99 for not recorded)*	______
103	Did the woman come to this hospital for routine labor and delivery or due to a complication?	Routine labor…………………….1<br><br>Complication…………………….2
104	Did the woman come to the hospital from home, or was she referred by the traditional birth attendant or a health facility?	Home…………………………….1<br><br>Referred from<br>Traditional Birth Attendant…….2<br><br>Referred from Health Facility....3<br><br>Other…………………………….4<br>(specify)___________________<br><br>*(If response is not "health facility", skip to 201)*
105	If the woman was referred from another health facility, what type of facility was it? *(write in)*	________________________________

200	Case Time Flow	Time (hh/mm)	AM or PM	Date (dd/mm)
201	Regarding this case…at what time did the woman arrive to the ward?	:	a.m.<br>p.m.	/
202	At what time was the woman *first* seen by a provider?	:	a.m.<br>p.m.	/
203	At what time, and on which date, was the complication resulting in the cesarean *first* detected?	:	a.m.<br>p.m.	/
204	What is the cadre of the provider who first noted the complication and need for cesarean? (*write the cadre*)	____________________________		

200	Case Time Flow	Time (hh/mm)	AM or PM	Date (dd/mm)
205	At what time, and on which date,….<br><br>…was the Surgical Provider first notified/told of the complication and need for cesarean?	:	a.m.<br>p.m.	/
206	(Continued… At what time, and on which date,….)<br><br>…did the Surgical Provider decide that cesarean was required?	:	a.m.<br>p.m.	/
207	…was the Anesthesia Provider, notified of the complication and need for cesarean?	:	a.m.<br>p.m.	/
208	…was the abdominal incision made?	:	a.m.<br>p.m.	/
209	… was the baby born?	:	a.m.<br>p.m.	/
210	… was the woman moved out of the operating theater to the post-surgical area?	:	a.m.<br>p.m.	/
211	… was the woman moved to the postpartum ward?	:	a.m.<br>p.m.	/

SECTION B – Observation of Cesarean Surgery

Please circle the response that corresponds with your observation. Few questions have an option of "not applicable" or N/A. This should be used rarely, when the item can not be done.

300	Initial assessment	Response
301	Does the provider perform a rapid initial assessment and institute emergency measures if necessary?	Yes …………………….1<br>No…………………......0
302	Does the surgical provider ask about the time of last fluid/solid intake?	Yes …………………….1<br>No…………………......0
303	Does the provider review the woman's chart?	Yes …………………….1<br>No…………………......0
304	Does anyone mobilize the surgical / operative theatre team?	Yes …………………….1<br>No…………………......0
305	Does anyone mobilize anesthesia?	Yes …………………….1<br>No…………………......0
306	Does anyone mobilize the pediatric team?	Yes …………………….1<br>No…………………......0

400	Physical exam	Response
401	Does the provider check blood pressure and pulse?	Yes ……………………1<br>No………………….......0
402	Does the provider check the temperature?	Yes ……………………1<br>No………………….......0
403	Does the surgical provider note urine output?	Yes ……………………1<br>No………………….......0
404	Does the surgical provider listen to lungs and heart?	Yes ……………………1<br>No………………….......0
405	Does the surgical provider palpate abdomen and uterus?	Yes ……………………1<br>No………………….......0
406	Does the surgical provider confirm presentation of fetus and whether the gestation is singleton vs. multiple?	Yes ……………………1<br>No………………….......0
407	Does the surgical provider confirm whether fetus is alive?	Yes ……………………1<br>No………………….......0
408	Does the surgical provider perform cervical exam if indicated?	Yes ……………………1<br>No………………….......0<br>N/A……………………99

500	Preparation for anesthesia and surgery	Response
501	Has an IV line been inserted?	Yes ……………………1<br>No………………….......0<br>*(If no, skip to 504)*
502	If yes, do the providers bolus…<br>…500 cc of IV fluids for general anesthesia	Yes ……………………1<br>No………………….......0<br>N/A……………………99
503	…1000cc of crystalloid fluids for regional anesthesia	Yes ……………………1<br>No………………….......0<br>N/A…………………...99
504	Does any provider…<br>…clean vulva?	Yes ……………………1<br>No………………….......0
505	…insert sterile catheter?	Yes ……………………1<br>No………………….......0
506	…give the woman an enema?	Yes ……………………1<br>No………………….......0
507	…shave the woman’s abdomen?	Yes ……………………1<br>No………………….......0

500	Preparation for anesthesia and surgery	Response		
508	…shave the woman's pubic hair?	Yes ……………………1<br>No………………….......0		
509	Does any provider give…<br>…Magnesium trisilicate 300 mgs	Yes ……………………1<br>No………………….......0		
510	…Sodium citrate 30 cc 0.3 molar	Yes ……………………1<br>No………………….......0		
511	…Ranitidine 150 mgs PO or 50 mgs IV	Yes ……………………1<br>No………………….......0		
512	Does any provider roll the patient 15 degrees to the left and place a pillow under the right hip to decrease risk of supine hypotension OR tilt the entire operating table to the left?	Yes ……………………1<br>No………………….......0		
513	Was the fetal heart rate assessed before beginning skin prep and surgery?	Yes ……………………1<br>No………………….......0<br>N/A……………………..99		
514	Time fetal heart rate was obtained	:	a.m.<br>p.m.	/
515	Is pulse oximetry machine checked before the start of the case?	Yes ……………………1<br>No………………….......0<br>N/A……………………..99		
516	Type of anesthesia used	General …………………1<br>Spinal ………..…………..2<br>Ketamine ……...………..3<br>Local …………..………..4<br>Ether……………………..5<br>Combination……………..6<br>*Specify*________________		
517	Ask Q517 – Q520 ONLY if general anesthesia was used.<br>If used other type of anesthesia, skip to Q601.<br><br>…Is the patient intubated?	Yes ……………………1<br>No………………….......0<br>N/A…………………...99		
518	…Does the anesthesia provider auscultate the chest to confirm that air is entering both lungs equally?	Yes ……………………1<br>No………………….......0<br>N/A…………………..99		
519	…Does the anesthesia provider auscultate over the stomach and ensure absence of air entry into the stomach?	Yes ……………………1<br>No………………….......0<br>N/A…………………..99		
520	…Does the surgical provider check that the anesthesia has taken full effect by speaking with anesthesia provider and gently pinching abdominal skin with a forceps or clamps?	Yes ……………………1<br>No………………….......0<br>N/A……………………..99		

Continued…

600	Cesarean Practice – From Beginning to Before Incision	Response
601	Does the surgical provider put on appropriate scrub clothes?	Yes ……………………1<br>No……………………......0
602	Does the anesthesia provider put on appropriate scrub clothes?	Yes ……………………1<br>No……………………......0
603a	Does the surgical provider put on…<br>…a cap?	Yes ……………………1<br>No……………………......0
603b	Does the surgical provider put on…<br>…protective eyeglasses or shield?	Yes ……………………1<br>No……………………......0
603c	Does the surgical provider put on…<br>…a facemask?	Yes ……………………1<br>No……………………......0
603d	Does the surgical provider put on…<br>…shoe covers?	Yes ……………………1<br>No……………………......0
604a	Does the anesthesia provider put on…<br>…a cap	Yes ……………………1<br>No……………………......0
604b	Does the anesthesia provider put on…<br>…protective eyeglasses or shield?	Yes ……………………1<br>No……………………......0
604c	Does the anesthesia provider put on…<br>…a facemask?	Yes ……………………1<br>No……………………......0
604d	Does the anesthesia provider put on…<br>…shoe covers?	Yes ……………………1<br>No……………………......0
605	Does the surgical provider perform a surgical hand scrub?	Yes ……………………1<br>No……………………......0
606	Does the anesthesia provider perform a surgical hand scrub?	Yes ……………………1<br>No……………………......0
607	Does the surgical provider put on HLD/sterile gloves?	Yes ……………………1<br>No……………………......0
608	Does the anesthesia provider put on clean or HLD/sterile gloves?	Yes ……………………1<br>No……………………......0
609	Does any provider apply antiseptic solution to the abdomen?	Yes ……………………1<br>No……………………......0
610	Does a provider check function of surgical suctioning?	Yes ……………………1<br>No…………………........0<br>N/A…………………..99
611	Does the surgical provider attach electrocautery tip to sterile connectors and check function?	Yes ……………………1<br>No…………………........0<br>N/A…………………...99
612	Does any provider ensure that bladder catheter is draining urine into gravity bag?	Yes ……………………1<br>No…………………........0<br>N/A…………………...99

	Cesarean Practice – From Incision to End	Response		
613	Skin incision time	:	a.m.<br>p.m.	/
613	What type of skin incision is made?	Midline vertical……………1<br>Low transverse……….......0		
615	What type of uterine incision is made?	Midline vertical……………1<br>Low transverse……….......0		
616	Does the provider clamp and tie or cauterize arterial and venous bleeding points as encountered?	Yes ……………………1<br>No…………………........0<br>N/A…………………..99		
617	Does the surgical provider push or sharply dissect the bladder downward off the lower uterine segment?	Yes ……………………1<br>No……………………......0		
618	Does the surgical provider use a bladder retractor?	Yes ……………………1<br>No……………………......0		
619	Does the surgical provider confirm presentation and positioning of the fetus by manual palpation of the uterus?	Yes ……………………1<br>No……………………......0		
620	Does the surgical provider inform newborn care team of the color of the amniotic fluid?	Yes ……………………1<br>No……………………......0		
621	Does the surgical provider suction the newborn's mouth and nose when delivered with bulb suction?	Yes ……………………1<br>No……………………......0<br>N/A…………………..99		
622	Does the surgical provider use DeLee suction apparatus if meconium stained fluid observed?	Yes ……………………1<br>No…………………........0<br>N/A…………………..99		
623	After delivery of the newborn, which uterotoic was the woman given, if any?	None………………………0<br>Oxytocin ……………..……1<br>Misoprostol ………………2<br>Ergometrine ……......…..3		
624	What dose was the woman given? *(write dose or 0 for "none")*	________________________<br>(specify units)		
625	If ergometrine is given, does the provider obtain the woman's blood pressure to ensure it is not elevated?	Yes ……………………1<br>No…………………........0<br>N/A…………………..99		
626	Does the surgical provider hand newborn to newborn care team while maintaining sterility of gloves and gown or change gloves if sterility is breeched?	Yes ……………………1<br>No…………….….......0		
627	After clamping the cord, does the woman receive prophylactic antibiotics?	No………………….................0<br>Yes, ampicillin 2 grams IV…. 1<br>Yes, other……………………2<br>(specify)__________________		

	Cesarean Practice – From Incision to End	Response
628	Does the provider deliver the placenta by…<br><br>…Controlled traction on the cord?	Yes ……………………1<br>No……………………......0
629	…Manual extraction?	Yes ……………………1<br>No……………………......0
630	…Both?	Yes ……………………1<br>No……………………......0
631	Does the provider inspect placenta for completeness or abnormalities?	Yes ……………………1<br>No……………………......0
632	Does the provider explore the pelvis to remove blood clots from pelvis?	Yes ……………………1<br>No……………………......0
633	Does the provider look for any extension of the uterine incision into the cervix/upper vagina?	Yes ……………………1<br>No……………………......0
634	Does the provider check the bladder for injury and repair injury if necessary?	Yes ……………………1<br>No……………………......0
635	Does the provider close the peritoneum?	Yes ……………………1<br>No……………………......0
636	Did the provider recheck the uterus to make sure it is firm?	Yes ……………………1<br>No……………………......0
637	Did the woman receive a tubal ligation during surgery?	Yes ……………………1<br>No……………………......0<br>*(If no, skip to 639)*
638	…if yes, did she request the tubal ligation?<br>*(please ask the providers or observe in chart)*	Yes ……………………1<br>No……………………......0<br>N/A…………………..99
639	Was a final sponge and instrument count done?	Yes ……………………1<br>No……………………......0
640	Does any provider apply a sterile dressing?	Yes ……………………1<br>No……………………......0
641	Does any provider apply water-occlusive covering over the dressing?	Yes ……………………1<br>No……………………......0
642	Does the provider ensure that urine is flowing into urine bag?	Yes ……………………1<br>No……………………......0<br>N/A…………………..99
643	In your opinion, did the client have adequate anesthesia throughout cesarean section?	Yes ……………………1<br>No……………………......0
644	Were the blood pressure and pulse measured at least every 15 minutes during the procedure?	Yes ……………………1<br>No……………………......0

	Cesarean Practice – From Incision to End	Response
645	How often were the blood pressure and pulse measured during the procedure?	Never……………………0<br>Once……………………..1<br>2 – 5 times………………2<br>6 or more times…………3
646	Was the airway monitored and respirations/pulse oximeter assessed?	Yes ……………………1<br>No……………………......0
647	If the woman was intubated, does the anesthesia provider confirm that the client is ready for extubation?	Yes ……………………1<br>No……………………......0<br>N/A…………………..99

SECTION C – Observation of Post-Operative and Newborn Care

Please circle the response that corresponds with your observation. Few questions have an option of "not applicable" or N/A. This response should be used rarely, only when the item can not be done.

Observe the following items in the first one-hour period of post-operative care for the mother.

700	Immediate Post-Operative Care - Observe for 1 hour	Response
701	In the first hour of the mother's recovery, how often (how many times) is…<br><br>… level of alertness checked?	_______ times
702	… B/P checked?	_______ times
703	… heart rate checked?	_______ times
704	… breathing checked?	_______ times
705	… temperature checked?	_______ times
706	… perineum checked for bleeding?	_______ times
707	… abdomen checked?	_______ times
708	… urine bag checked for urine flow?	_______ times

Continued…

Please observe the following items in the two-hour post-operative care period for the mother.

800	Immediate Post-Operative Care - Observe for 2 hours	Response
801	Is the IV maintained during recovery?	Yes ……………………1<br>No……………………......0
802	Are antibiotics continued for the postoperative period?	Yes ……………………1<br>No……………………......0
803	Was the woman given blood during or shortly after the cesarean?	Yes ……………………1<br>No……………………........0<br>*(If no, skip to 804)*
804	If yes, was blood given as a routine measure or due to hemorrhage?	Routine ………………1<br>Hemorrhage…………..2
805	Is the woman ever left unattended in the first hour?	Yes ……………………1<br>No……………………......0
806	Does the surgical provider use antiseptic hand scrub or wash hands thoroughly with soap and water after the case?	Yes ……………………1<br>No……………………......0
807	Does the surgical assistant use antiseptic hand scrub or wash hands thoroughly with soap and water after the case?	Yes ……………………1<br>No……………………......0
808	Does the provider write an operation note?	Yes ……………………1<br>No……………………......0
809	Does the provider write postoperative management instructions?	Yes ……………………1<br>No……………………......0

Please observe the following items in the two-hour post-operative care period for the baby.

900	Immediate Newborn Care	Response
901	Immediately after birth, is the baby dried and wrapped in a dry blanket OR dried and placed under a warming light?	Yes …………………………………………1<br>No…………………..................................0<br>N/A………………………………………99
902	Is the baby slapped to stimulate breathing?	Yes …………………………………………1<br>No…………………..................................0
903	Is the baby rubbed to stimulate breathing?	Yes …………………………………………1<br>No…………………..................................0

900	Immediate Newborn Care	Response
904	What is the condition of the newborn immediately after birth?	Normal: crying or pink and alert with good tone ………………………………………….1<br>*(If Normal, skip to 1000)*<br><br>Limp and/or pink cyanotic………………….2<br>*(If Limp skip to 1100)*<br><br>Stillborn, Macerated………………………...3<br><br>Stillborn, Fresh…………………………….4<br>*(If stillborn, skip to 1200)*

Please observe the following items in the first <u>one-hour</u> period for the newborn.

1000	Normal Newborn – 1st hour	Response
1001	How often in the first hour is the baby's…<br><br>… heart rate checked?	_______ times
1002	… breathing checked?	_______ times
1003	… temperature checked by thermometer?	_______ times
1004	… temperature checked by touch?	_______ times
1005	Are antimicrobial drops placed in the baby's eyes?	Yes ………………………………………….1<br>No………………….................................0
1006	Is the baby left unattended at any time?	Yes ………………………………………….1<br>No………………….................................0
		<u>Skip to 1200</u>

1100	Limp or Cyanotic Newborn	Response
1101	Is the baby placed on her/his back on warm surface?	Yes ………………………………………….1<br>No………………….................................0
1102	Is the baby kept wrapped except for face and chest?	Yes ………………………………………….1<br>No………………….................................0
1103	Is the airway cleared before beginning further resuscitation?	Yes ………………………………………….1<br>No………………….................................0
1104	Is baby ventilated with bag and mask attached to oxygen?	Yes ………………………………………….1<br>No………………….................................0<br>*(If no, skip to 1006)*

1100	Limp or Cyanotic Newborn	Response
1105	If yes, what is the size of the mask?	________________<br>(specify units)
1006	Does the baby require intubation?	Yes ……………………………………….1<br>No………………….................................0
1007	Does the baby receive cardiac compressions?	Yes ……………………………………….1<br>No………………….................................0<br>*(If no, skip to 1009)*
1008	If yes, for how long did the baby receive compressions?	_____ minutes
1009	Is the mother (if awake) kept informed of condition of her baby?	Yes, informed ……………………………...1<br>No, not informed…………………………...2<br>N/A, mother not awake…………………....3
1010	If the baby is born alive and requires resuscitation, what is the outcome for the baby?	Alive and admitted to care of mother or normal newborn nursery ………………….1<br><br>Alive and admitted to special care for sick newborns……………………………….......2<br><br>Alive and transferred to another facility for special care……………………………......3<br><br>N/A, baby is dead…………………………..4

Please observe the following interpersonal items for the overall case.

1200	Overall Client Provider interaction	Response
1201	Does a member of the provider team explain the reason(s) for the cesarean to the woman and/or her companion/family?	Yes ……………………………1<br>*(If yes, skip to 1203)*<br>No…………………..................0
1202	If not, why were reasons not explained?	___________________________
1203	Did the surgical and/or anesthesia provider counsel the woman and/or companion/family on the risks and benefits of cesarean section and anesthesia?	Yes ……………………………1<br>No…………………..................0
1204	Did the surgical provider answer any questions of the woman and her companion/family?	Yes ……………………………1<br>No…………………..................0<br>N/A (No questions were asked).99
1205	Did a member of the provider team obtain signed written consent from client to proceed with cesarean section and anesthesia?	Yes ……………………………1<br>No…………………..................0

1200	Overall Client Provider interaction	Response
1206	Did the surgical provider keep the woman and/or her companion/family informed at all times?	Yes ……………………………1<br>No…………………................0
1207	Overall, did the team of providers maintain privacy for the woman including draping for procedures?	Yes ……………………………1<br>No…………………................0
1208	Overall, did the team of providers maintain confidentiality of information?	Yes ……………………………1<br>No…………………................0
1209	Overall, how respectful or disrespectful was the team of providers to the woman and/or her family?	Very respectful………………1<br>Somewhat respectful………..2<br>Somewhat disrespectful…….3<br>Very disrespectful……………4

END of Observation
