# Supplementary material for: Using direct clinical observation to assess the quality of cesarean delivery in Afghanistan: an exploratory study
Source: BMC Pregnancy Childbirth. 2014 May 27;14:176. doi: 10.1186/1471-2393-14-176 (PMC4062280; doi:10.1186/1471-2393-14-176)
Supplement: Additional file 2 — Afghanistan Emergency Obstetric and Newborn Care National Assessment Tool: Review of Unobserved Post-Cesarean Case. [file 1471-2393-14-176-S2.pdf]

Approved: 15APR10

Hospital: – National ID Number: ____________________

Study-generated ID for the Post-CS Case: ____________________

EmONC Needs Assessment in Afghanistan-Part III Cesarean Section
Jhpiego

Tool 4: Review of Unobserved Post-Cesarean Case

~~~~~~~~~~~~~~~~~~~~~~~~~~~~~~~~~~~~~

	Information about Data Collection	Response
1	Assessor Name /ID Number:	___________________________
2	Health Facility Type: *(circle response)*	DH(1), PH(2), RH(3), SH(4)
3	Today's Date: (mm/dd):	___/ ___
4	Date When CS was Done:	___/ ___
5	Study-generated Surgical Provider ID who did CS:	___________________________

Instructions to the Assessor: *.*

Please identify all the unobserved cesarean cases that occurred more than 12 hours ago but less than 60 hours ago. That is, review cases of women who had a cesarean within the last 60 hours (2.5 days) who have had at least 12 hours of post-operative care.

Randomly select cases to review according to the sampling plan in the study protocol. Fill in one form completely for each case.

The sections of this tool are organized according to the source of data specified.

- *Section A. Data from Records or Interview with Surgical Provider or Nurse*
- *Section B. Data from Records Only*
- *Section C. Data from Interview with Surgical Provider First or Records*
- *Section D. Data from the Interview with Surgical Provider Only*
~~~~~~~~~~~~~~~~~~~~~~~~~~~~~~~~~~~~~

SECTION A. Data from Records or Interview with Surgical Provider or Nurse

In Section A, please attempt to obtain the requested information from the case records. If the information is not available, ask the Surgical Provider or Nurse.

For each question:

- *write in the response or circle the number of the appropriate response, and*
- *at the far-right column, circle the number corresponding to the source of the data. (**RR**=Record Review, **NI**= Nurse Interview, **SI**=Surgical Provider Interview)*

100	Woman's Arrival to Hospital	Response	Source of Data
101	Regarding this case…did the woman come to this hospital for routine labor and delivery, or due to a complication?	Routine labor………………...1<br>Complication…………………2	RR NI SI<br>1 2 3
102	Did the woman come to the hospital from home, or was she referred by the traditional birth attendant or a health facility?	Home…………………………..1<br><br>Referred from<br>Traditional Birth Attendant…...2<br><br>Referred from Health Facility...3<br><br>Other……………………………4<br>(specify)__________________<br><br>*(If response is not "health facility", skip to 201)*	RR NI SI<br>1 2 3
103	If the woman was referred from a health facility, what type of facility was it?<br>*(write in)*	____________________________	RR NI SI<br>1 2 3

200	Overall Case Time Flow	Time (hh/mm)	AM or PM	Date (dd/mm)	Source of Data
201	Regarding this case…at what time was the woman admitted to the ward?	:	a.m.<br>p.m.	/	RR NI SI<br>1 2 3
202	At what time was the woman *first* seen by a provider?	:	a.m.<br>p.m.	/	RR NI SI<br>1 2 3

200	Overall Case Time Flow	Time (hh/mm)	AM or PM	Date (dd/mm)	Source of Data
203	At what time, and on which date, was the complication resulting in the cesarean *first* detected?	:	a.m.<br>p.m.	/	RR NI SI<br>1 2 3
204	What is the cadre of the provider who first noted the complication and need for cesarean? (*write the cadre*)	______________			RR NI SI<br>1 2 3
205	(Continued…At what <u>time</u>, and on which <u>date</u>,….)<br><br>…was the Surgical Provider first notified of the complication and need for CS?	:	a.m.<br>p.m.	/	RR NI SI<br>1 2 3
206	At what <u>time</u>, and on which <u>date</u>,….<br><br>…did the Surgical Provider decide that cesarean was required?	:	a.m.<br>p.m.	/	RR NI SI<br>1 2 3
207	… was the Anesthesia Provider notified of the complication and need for cesarean?	:	a.m.<br>p.m.	/	RR NI SI<br>1 2 3
208	… was the abdominal incision made?	:	a.m.<br>p.m.	/	RR NI SI<br>1 2 3
209	… was the baby born?	:	a.m.<br>p.m.	/	RR NI SI<br>1 2 3
210	… was the woman moved out of the operating theater to the post-surgical area?	:	a.m.<br>p.m.	/	RR NI SI<br>1 2 3
211	… was the woman moved to the postpartum ward?	:	a.m.<br>p.m.	/	RR NI SI<br>1 2 3

SECTION B. Data from Records Only

In Section B, please obtain the requested information from the case records only. This can include the patient medical record, delivery register, or operating theater register. If the information is not found in the records, then indicate a response of "No" for "not recorded." For each question, write in the response or circle the number of the appropriate response.

300	Operative Care	Response
301	What is the age of the mother? *(write 99 if not recorded)*	______
302	What is the parity of the mother? *(write 99 if not recorded)*	______

300	Operative Care	Response
303	Is there a consent form signed by either the woman or her family before her cesarean section?	Yes ……………………1<br>No…………………......0
304	Is there written documentation of verbal consent?	Yes ……………………1<br>No…………………......0
305	Was the fetal heart beat recorded in the operating theater?	Yes ……………………1<br>No…………………......0
306	What type of anesthesia was used for this case?	General ………………1<br>Spinal ……….………..2<br>Ketamine ……..……..3<br>Local ………..……..4<br>Ether…………………...5<br>Combination……….....6<br>*(specify):*<br>________________
307	Was an indwelling urinary catheter placed?	Yes ……………………1<br>No…………………......0
308	Was the woman intubated?	Yes ……………………1<br>No…………………......0
309	What type of skin incision was made?	Vertical …………….....1<br>Low transverse ……...2<br>T-incision ……..…….3
310	What type of uterine incision was made?	Vertical ……………….1<br>Low transverse ………2<br>T-incision ……..……..3
311	After delivery of the newborn, which uterotoic was the woman given, if any?	None…………………..0<br>Oxytocin ……………...1<br>Misoprostol …………..2<br>Ergometrine ………....3
312	What dose was the woman given? *(write dose or 0 for "none")*	____________________<br>(specify units)
313	After clamping the cord, which prophylactic antibiotics did the woman receive, if any?	None……………………0<br>Ampicillin 2 grams IV ….1<br>Other …………….2<br>*(specify):*____________
314	What dose was the woman given? *(write dose or 0 for "none")*	____________________<br>(specify units)
315	Was the peritoneum closed?	Yes ……………………1<br>No…………………......0
316	How many minutes was the operative time?	_______ minutes
317	How many times were the woman's vitals signs recorded during the surgery?	_______ times

Continued…

**400**	**Operative Care-Newborn**	**Response**
400	Did the newborn require bag and mask ventilation?	Yes ………………………………..1<br>No…………………………………....0
401	Did the newborn require intubation?	Yes ………………………………..1<br>No…………………………………....0
402	Did the newborn require cardiac compressions?	Yes ………………………………..1<br>No…………………………………....0
403	Was the baby born alive?	Yes ………………………………..1<br>No…………………………………....0<br>*(if no, skip next 405)*
404	Was the baby…	Admitted to normal newborn nursery or to mother's care mother?.........1<br><br>Admitted to special care for sick newborns?…………………………2<br><br>Transferred to another facility for special care?...............................3<br><br>*(Skip to 501)*
405	Was the baby…	Macerated stillborn?…………......1<br>Fresh stillborn?………………......2<br>Died after birth?……………..….3

**500**	**Determination of 12-Hour Post-Operative Period**	**Time and Date**		
501	*Please transpose the time and date of the cesarean from question 209.*	:	a.m.<br>p.m.	/
502	*Please add 12 hours to the cesarean time in the question above, to be clear of the first 12 hour-period after CS.*	:	a.m.<br>p.m.	/
503	*What time are you collecting this information (what time is it right now)?*	:	a.m.<br>p.m.	/

Continued…

For the questions below, write in the number of times each item is done. If the information was not recorded, circle 99. Please note that you should only count the number of occurrences in the first 12 hours after the cesarean surgery was completed.

600	Post-CS care within 12 hours (1)	Response
601	In the first 12 hours after the CS operation, how many times were the following checked?<br><br>…Blood pressure	Number of times ______<br>Not Recorded…………99
602	…Pulse	Number of times ______<br>Not Recorded…………99
603	…Respiration	Number of times ______<br>Not Recorded…………99
604	…Temperature	Number of times ______<br>Not Recorded…………99
605	… Perineal pad for bleeding	Number of times ______<br>Not Recorded…………99
606	…Abdomen	Number of times ______<br>Not Recorded…………99
607	…Urine measured	Number of times ______<br>Not Recorded…………99
608	…Oral intake measured	Number of times ______<br>Not Recorded…………99
609	…IV fluids checked/replaced	Number of times ______<br>Not Recorded…………99
610	In the first 12 hours after the CS operation, how many times…<br>…has the mother's pain level been assessed?	Number of times ______<br>Not Recorded…………99
611	…has pain medicine been given to the mother?	Number of times ______<br>Not Recorded…………99
612	…has the mother been out of bed (ambulated)?	Number of times ______<br>Not Recorded…………99

600	Post-CS care within 12 hours (1)	Response
613	…has the baby breastfed?	Number of times ______<br>Not Recorded…………99

Continued…

700	Post-CS care within 12 hours (2)	Response
701	How many hours after the birth…<br><br>…was the mother given oral fluids (first time)?	Number of hours after birth: ____<br>Not Recorded…………99
702	…was the mother given solids?	Number of hours after birth: ____<br>Not Recorded…………99
703	…was the IV removed?	Number of hours after birth: ____<br>Not Recorded…………99
704	…was the urinary catheter removed?	Number of hours after birth: ____<br>Not Recorded…………99

SECTION C. Data from Interview with Surgical Provider First or Records

In Section C, please obtain the requested information from the Surgical Provider. If the Surgical Provider is not available, ask if the Surgical Provider may be available later. If the Surgical Provider is not available at any time, obtain this information from the records. At the far-right column, circle the number corresponding to the source of the data (RR=Record Review, SI=Surgical Provider Interview).

800	CS Indication	Response	Source of Data
800a	Was the Surgical Provider available for interview?	Yes …………………1<br>No………………....0	
801	What is the **indication** for the surgery?<br><br>…history of prior cesarean section or fistula	Yes …………………1<br>No………………....0	RR SI<br>1 3
802	…placenta previa	Yes …………………1<br>No………………....0	RR SI<br>1 3

800	CS Indication	Response	Source of Data
803	…vaginal hemorrhage	Yes …………………1<br>No……………….....0	RR SI<br>1 3
804	…suspected uterine rupture		RR SI<br>1 3
805	… malpresentation of the fetus	Yes …………………1<br>No……………..…...0<br>(*If no, skip to 807)*	RR SI<br>1 3
806	What type of fetal malpresentation was it?	Breech……………..1<br>Transverse…………2<br>Face…………………3<br>Brow.………………4	RR SI<br>1 3
807	(Continued…What is the **indication** for the surgery?)<br><br>…multiple gestation	Yes …………………1<br>No………………….0<br>(*If no, skip to 809)*	RR SI<br>1 3
808	What was the multiple gestation?	Twins with Breech Presentation……….1<br>Triplets or greater…2	RR SI<br>1 3
809	… fetal distress by abnormal fetal heart beat	Yes …………………1<br>No……………….....0	RR SI<br>1 3
810	… meconium stained amniotic fluid	Yes …………………1<br>No……………….....0	RR SI<br>1 3
811	… preeclampsia/eclampsia	Yes …………………1<br>No……………….....0	RR SI<br>1 3
812	… failure to progress in labor/obstructed labor	Yes …………………1<br>No……………….....0	RR SI<br>1 3
813	… other: please specify ______________________	Yes …………………1<br>No……………….....0	RR SI<br>1 3
814	From the above list, which ONE indication was the *main indication* for the CS?<br><br>*Write in name of indication and circle question number above: (Example: 812. Pre-eclampsia/eclampsia)*	___________________	RR SI<br>1 3

900	Practices Prior to CS	Response	Source of Data

900	Practices Prior to CS	Response	Source of Data
901	Was a partogram used?<br><br>*(If the Surgical Provider is unsure whether it was used, please review the record.)*	Elective CS, therefore no partogram……...0<br><br>Partogram used…….1<br><br>Partogram not used..2	RR SI<br>1 3
902	Was labor augmented?	Yes …………………1<br>No…………………...0<br>*(If no, skip to 904)*	RR SI<br>1 3
903	How was labor augmented?	Amniotomy ………..1<br>Oxytocin ………….2<br>Misoprostol …..…..3<br>Other (specify)…….4:<br>________	RR SI<br>1 3
904	Was assisted vaginal delivery with vacuum attempted?	Yes …………………1<br>No………………....0	RR SI<br>1 3
905	Was assisted vaginal delivery with forceps attempted?	Yes …………………1<br>No………………....0	RR SI<br>1 3
906	Was the fetus dead?	Yes …………………1<br>No…………………...0<br>*(If no, skip to 1001)*	RR SI<br>1 3
907	If the fetus was dead, was craniotomy attempted?	Yes …………………1<br>No………………....0	RR SI<br>1 3

SECTION D. Data from the Surgical Provider Only

In Section D, ask the questions to the Surgical Provider only. If the Surgical Provider is not available, leave this section blank.

1000	Urgency of CS	Response
1001	In whose interest was the CS done?	Mother……………………………………...1<br>(*If Mother, skip to 1003)*<br><br>Child……………………………………..2<br>(*If Child, skip to 1003*)<br><br>Both………………..................................3
1002	If both, for whom was the CS more urgent?	Mother……………………...................1<br><br>Child……………………………………..2

1003	Was the CS unscheduled (due to an emergency) or scheduled (not due to an emergency)?	Unscheduled/emergency…………………1<br><br>Scheduled/not emergency………………2
1004	Did the CS happen due to…	a problem identified during pregnancy?...1<br><br>a problem identified during labor?……….2<br><br>preference or convenience (patient choice, doctor schedule, etc.)?……………………3
	END	
