# Supplementary material for: Using direct clinical observation to assess the quality of cesarean delivery in Afghanistan: an exploratory study
Source: BMC Pregnancy Childbirth. 2014 May 27;14:176. doi: 10.1186/1471-2393-14-176 (PMC4062280; doi:10.1186/1471-2393-14-176)
Supplement: Additional file 3 — Afghanistan Emergency Obstetric and Newborn Care National Assessment Tool: Interview of Anesthesia and Surgical Providers. [file 1471-2393-14-176-S3.pdf]

Approved: 15APR10

Hospital: National ID Number: ____________________

Study-generated Surgeon ID: ____________________

Facility Type (*Circle*): DH(1), PH(2), RH(3), SH(4)

Today's Date: (mm/dd): ___/ ___

EmONC Needs Assessment in Afghanistan-Part III Cesarean Section (CS)
Jhpiego

Tool 5: Self-Administered Questionnaire of Surgical Provider's Experience and Practice

~~~~~~

Instructions to the Surgical Provider: *Your responses to the questions below are important. The questions ask about your training and experience; usual cesarean practices, anesthesia, and post-operative care. Please complete this questionnaire and return it to the Assessor today. Please write in or circle your response to each question. Thank you.*

100	Training and Experience	Response
101	In what year were you *first* trained to do cesarean section? *Write in the year:*	— — — —
102	In what year was your *most recent* training related to cesarean section? *Write in the year:*	— — — —
103	At what institution was your *most recent* training related to cesarean section? *Write in the institution and location:*	Institution: __________________<br>District: __________________<br>Province: __________________
104a	Of all the training you received related to cesarean section, have you received:<br>…formal, *pre-service* training? *(circle response)*	Yes ………………….1<br>No…………………...0
104b	…formal, *in-service* training?	Yes ………………….1<br>No…………………...0
104c	… *informal, on-the-job* training?	Yes ………………….1<br>No…………………...0
105	When was the last time you did a cesarean section? *Write in month, day, and year (mm/dd/yr):*	Month: __________<br>Day: ___________<br>Year:____________
106	How many cesarean sections have you done in the last six (6) months? *Write in number:*	______
~~~~~~

100	Training and Experience	Response
107	How would you rate your level of confidence in doing cesarean section?<br>*(circle one response)*	**Not at all** confident.................1<br>**A little** confident……………….2<br>**Fairly** Confident………………..3<br>**Very** Confident………….........4
108	How would you rate your level of confidence in teaching cesarean section to other providers?<br>*(circle one response)*	**Not at all** confident.................1<br>**A little** confident……………….2<br>**Fairly** Confident………………...3<br>**Very** Confident…………..........4

Next, we would like to ask you about the way you usually perform cesareans.

200	Usual CS Practices	Response
201	Is it your usual practice to request or administer an enema to empty the bowel in preparation for cesarean section?	Yes …………………1<br>No…………………...0
202	Is it your usual practice to shave the lower abdomen and pubic hairs in preparation for cesarean section?	Yes …………………1<br>No…………………...0
203a	Is it your usual practice to deliver the woman by cesarean section if the woman has the following condition?<br>… eclampsia	Yes …………………1<br>No…………………...0
203b	… short primigravida	Yes …………………1<br>No…………………...0
203c	… women with one previous cesarean section	Yes …………………1<br>No…………………...0
203d	… women with two previous cesarean sections	Yes …………………1<br>No…………………...0
203e	… women who have secondary arrest of labor with good contractions	Yes …………………1<br>No…………………...0
203f	… women who have meconium stained liquid in early labor	Yes …………………1<br>No…………………...0
204	Is it your usual practice to do cesarean sections using…	General Anesthesia……...1<br>Regional Anesthesia ……0
205	Is a laryngeal mask usually in your operating theatre?	Yes …………………1<br>No…………………...0
206	Is it your usual or routine practice to do the following?:<br>… transfuse one unit of blood during or shortly after cesarean	Yes …………………1<br>No…………………...0
207	…open the abdomen by transverse incision	Yes …………………1<br>No…………………...0
208	…open the uterine cavity with a vertical incision	Yes …………………1<br>No…………………...0

200	Usual CS Practices	Response
209	…ask a provider to give oxytocin to the mother after the delivery of the baby	Yes …………………1<br>No…………………...0
210	…perform tubal ligation after the mother's third (3rd) cesarean	Yes …………………1<br>No…………………...0
211	Is it your usual or routine practice to do the following?:<br>…give a single dose of antibiotics to the mother after the cord is clamped	Yes …………………1<br>No…………………...0
212	…close the peritoneum	Yes …………………1<br>No…………………...0

300	Post-operative Care	Response
301	Is it your usual or routine practice in post-operative care to…:<br>…restrict oral intake for 24 hours?	Yes …………………1<br>No…………………...0
302	…restrict ambulation for 24 hours?	Yes …………………1<br>No…………………...0
303	…prescribe antibiotics for 5 to 7 days in all cesarean cases?	Yes …………………1<br>No…………………...0
304	…encourage breastfeeding as soon as client has recovered from anesthesia?	Yes …………………1<br>No…………………...0
305	…discharge patients on the third day after cesarean, if all is well?	Yes …………………1<br>No…………………...0

Thank you for taking the time to complete this questionnaire.
