# Supplementary material for: Using direct clinical observation to assess the quality of cesarean delivery in Afghanistan: an exploratory study
Source: BMC Pregnancy Childbirth. 2014 May 27;14:176. doi: 10.1186/1471-2393-14-176 (PMC4062280; doi:10.1186/1471-2393-14-176)
Supplement: Additional file 4 — Afghanistan Emergency Obstetric and Newborn Care National Assessment Tool: Self-Administered Questionnaire of Surgical Provider’s Experience and Practice. [file 1471-2393-14-176-S4.pdf]

Approved: 15APR10

Hospital: – National ID Number: ____________________

Study-generated ID for Surgical Provider: ____________________

Study-generated ID for the CS Case: ____________________

EmONC Needs Assessment in Afghanistan-Part III: Cesarean Section (CS)
Jhpiego

Tool 3: Interview on the Observed CS with Anesthesia and Surgical Providers

~~~~~~

	Information about Data Collection	Response
1	Assessor Name /ID Number:	
2	Health Facility Type: *(circle response)*	DH(1), PH(2), RH(3), SH(4)
3	Today's Date: (mm/dd):	___/ ___

Instructions: Please administer this Tool 2 after the Tool 3 Observation. Interview the Anesthesia Provider (in Section A) and the Surgical Provider (in Section B) who performed the observed cesarean. For each question, circle the appropriate response or write in the blank space.

Section A: Questions for Anesthesia or Surgical Provider who did Anesthesia

100	Anesthesia Used	Response
101	What type of anesthesia was used for this case?	General …………………1<br>Spinal ……….…………..2<br>Ketamine ……..………..3<br>Local …………..………..4<br>Ether……………………..5<br>Combination………….....6<br>*Specify*________________
102	*For what reason* did you choose this method of anesthesia? *(write in response)*<br>____	
	Thank you for your time. *[**END of interview with Anesthesia Provider**]*	
~~~~~~

Section B: Questions for Surgical Provider

200	CS Indication	Response
201	What is the **indication** for the surgery?<br><br>…history of prior cesarean section or fistula	Yes ………………1<br>No………………...0
202	(Continued…What is the **indication** for the surgery?)<br><br>…placenta previa	Yes …………………1<br>No…………………..0
203	…vaginal hemorrhage	Yes …………………1<br>No…………………..0
204	…suspected uterine rupture	Yes …………………1<br>No…………………..0
205	… malpresentation of the fetus	Yes …………………1<br>No…………………..0<br>(*If no, skip to 207)*
206	What type of fetal malpresentation was it?	Breech……………….1<br>Transverse………….2<br>Face…………………3<br>Brow…………………4
207	…multiple gestation	Yes …………………1<br>No…………………..0<br>(*If no, skip to 209)*
208	What was the multiple gestation?	Twins with Breech Presentation……….1<br>Triplets or greater…2
209	… fetal distress by abnormal fetal heart beat	Yes …………………1<br>No…………………..0
210	… meconium stained amniotic fluid	Yes …………………1<br>No…………………..0
211	… preeclampsia/eclampsia	Yes …………………1<br>No…………………..0
212	… failure to progress in labor/obstructed labor	Yes …………………1<br>No…………………..0
213	… other: please specify ______________________	Yes …………………1<br>No…………………..0
214	From the above list, which ONE indication was the <u>*main*</u> *indication* for the CS?<br>*Write in name of indication and circle question number above: (Example: 812. Pre-eclampsia/eclampsia)*	__________________

Continued…

300	Practices Prior to CS	Response
301	Prior to the cesarean, was a partogram used?<br>*(If the Surgical Provider is unsure whether it was used, please review the record.)*	Elective CS, therefore no partogram……..........0<br>Partogram used……..1<br>Partogram not used…2
302	Was labor augmented?	Yes …………………1<br>No…………………..0<br>*(If no, skip to 304)*
303	How was labor augmented?	Amniotomy ………..1<br>Oxytocin …………...2<br>Misoprostol ….…….3<br>Other (specify)…….4<br>________
304	Was assisted vaginal delivery with vacuum attempted?	Yes …………………1<br>No…………………..0
305	Was assisted vaginal delivery with forceps attempted?	Yes …………………1<br>No…………………..0
306	Was the fetus dead?	Yes …………………1<br>No…………………..0<br>*(If no, skip to 401)*
307	If the fetus was dead, was craniotomy attempted?	Yes …………………1<br>No…………………..0

400	Urgency of CS	Response
401	In whose interest was the CS done?	Mother………………………………..........1<br>(*If Mother, skip to 403)*<br>Child……………………………………….2<br>(*If Child, skip to 403*)<br>Both………………...................................3
402	If both, for whom was the CS more urgent?	Mother……………………....................1<br>Child……………………………………........2
403	Was the CS unscheduled (due to an emergency) or scheduled (not due to an emergency)?	Unscheduled/emergency……………….1<br>Scheduled/not emergency…………….2
404	Did the CS happen due to a…	problem identified during pregnancy?......1<br>problem identified during labor?…………2<br>preference or convenience (patient choice, doctor schedule, etc.)?……………………3
